# Overview of COVID-19 Infection, Treatment, and Prevention in Children

**DOI:** 10.3390/jcm13020424

**Published:** 2024-01-12

**Authors:** Carol M. Kao

**Affiliations:** Department of Pediatrics, Division of Pediatric Infectious Diseases, Washington University School of Medicine, 660 S. Euclid Avenue, St. Louis, MO 63110, USA; kaoc@wustl.edu

**Keywords:** COVID-19, pediatrics, MIS-C, long COVID, COVID-19 vaccines

## Abstract

Coronavirus disease 2019 (COVID-19), caused by the novel respiratory virus—severe acute respiratory syndrome coronavirus 2 (SARS-CoV-2)—was declared a global pandemic by the World Health Organization on 11 March 2020. Since then, substantial gains have been made in our understanding of COVID-19 epidemiology, disease presentation, and management. While children tend to have less severe disease courses compared to adults, children can still develop severe COVID-19 infections, particularly in those with underlying medical conditions such as obesity, chronic lung disease, or prematurity. In addition, children are at risk of severe complications of COVID-19 infection, such as multisystem inflammatory syndrome in children (MIS-C) or long COVID. The case definitions of MIS-C and long COVID have continued to evolve with the increased understanding of these new entities; however, improved methods of diagnosis and determination of the optimal management are still needed. Furthermore, with the continued circulation of SARS-CoV-2 variants, there remains a need for clinicians to remain up-to-date on the latest treatment and prevention options. The purpose of this review is to provide an evidence-based review of what we have learned about COVID-19 in children since the start of the pandemic and how best to counsel children and their families on the best methods of prevention.

## 1. Introduction

Our understanding of severe acute respiratory syndrome coronavirus 2 (SARS-CoV-2) infections in children has increased considerably since the start of the COVID-19 pandemic. Generally, children have milder disease courses compared to adults and make up a small proportion of the overall hospitalizations and deaths due to COVID-19 [1]. However, children can still develop severe COVID-19 infection requiring hospitalization or post-infectious complications, such as multisystem inflammatory syndrome in children (MIS-C) or long COVID-19 [2]. Furthermore, there remains a paucity of pediatric-specific data on the optimal management and prevention of COVID-19. As SARS-CoV-2 variants of concern continue to circulate worldwide, there remains a pressing need for clinicians to remain up-to-date on COVID-19 epidemiology, treatment, and prevention strategies, such as the recommendations for updated COVID-19 vaccines. The purpose of this review is to provide an evidence-based review of what we have learned about COVID-19 in children since the start of the pandemic. 

## 2. COVID-19 Infections in Children

Early in the COVID-19 pandemic, children somewhat surprisingly made up a small proportion of the overall cases and hospitalizations due to COVID-19 infections [3]. However, in retrospect, these rates were likely an under-representation of the true burden of pediatric infection, particularly given limited diagnostic testing at the time. We have since learned that children are more likely to remain asymptomatic or be minimally symptomatic compared to adults and, thus, are less likely to have been tested [4]. In addition, widespread mitigation measures, such as school closures, were in place at the time, limiting community spread [3]. Subsequent transmission studies from large exposures or household studies have shown that children are as likely to become infected as adults and are able to transmit the virus to others [5,6]. However, with appropriate precautions, COVID-19 transmission from children, such as in in-person school settings, has been rare, particularly amongst vaccinated students [7,8,9,10].

Children with COVID-19 are frequently asymptomatic or minimally symptomatic, particularly younger children [11]. In a prospective surveillance study of children aged 0 to 4 years and their household members, young children with COVID-19 infection were more likely to be asymptomatic compared to their older siblings or adult household members [11]. Among symptomatic individuals in the study, young children also developed fewer symptoms. Symptoms of COVID-19 infection in children can be similar to other respiratory viral infections, and testing for SARS-CoV-2 is needed to confirm the diagnosis [12]. The most commonly reported symptoms of COVID-19 in children are fever, cough, myalgias, sore throat, headache, and malaise [13]. In one systematic review, fever was the only presenting symptom in 23% of infected children [14]. Less common symptoms include shortness of breath, gastrointestinal symptoms such as nausea or vomiting, neurologic symptoms, or rash [15]. When chest imaging is obtained, the majority of patients have normal chest X-rays [15]. However, the most common findings on chest CT scans when obtained are diffuse ground-glass opacities, consolidation, and pneumonia [14]. 

A subset of children can develop severe COVID-19 infection, resulting in hospitalization, need for supplemental oxygen, or even death [16]. As of 4 November 2023, there have been approximately 90,000 pediatric hospitalizations due to COVID-19 in the US [1]. The estimated incidence of pediatric intensive care unit (ICU) admission for COVID-19 is 12 per 100,000 children in the US [16]. COVID-19 was the leading infectious cause of death in US children between 2020 and 2022, although only associated with 2% of all-cause deaths [17]. Children with underlying medical conditions such as obesity, diabetes, chronic lung disease, neurologic disorders, prematurity, and cardiovascular disease are at increased risk for severe disease [16]. In addition, children at extremes of age (e.g., young infants and adolescents) are also at increased risk for severe COVID-19 [16]. Infants less than one year of age accounted for 20.3% of pediatric hospitalizations for severe COVID-19 in one multicenter surveillance study, of which 53.1% required supplemental oxygen, 14.8% required non-invasive mechanical ventilation or high-flow nasal cannula, 11.7% received mechanical ventilation, and 1.6% required extracorporeal membrane oxygenation (ECMO) [18]. The identification of novel inborn errors of immunity in children with severe COVID-19 infection is also an area of active research [19,20]. 

Amongst children with severe COVID-19 enrolled in the multicenter Overcoming COVID-19 Public Health Surveillance Registry study, non-invasive mechanical ventilation was required in 24.4% of cases, invasive mechanical ventilation in 30.2%, vasopressor use in 19.9%, and ECMO in 2.9% [21]. Critically ill children with COVID-19 had longer lengths of intensive care unit (ICU) stay and hospital lengths of stay than children hospitalized in an ICU for influenza [21]. Cardiovascular manifestations and complications related to COVID-19 infection are uncommon but have been described, including myocardial injury, arrhythmias, acute coronary syndrome, and venous thromboembolism [22]. Severe neurologic involvement has also been reported, including severe encephalopathy, stroke, central nervous system infection/demyelination, Guillain-Barre syndrome, and acute fulminant cerebral edema [23].

## 3. Multisystem Inflammatory Syndrome in Children (MIS-C)

In April 2020, clinicians in the United Kingdom first reported clusters of previously healthy children presenting with cardiovascular shock, fever, and Kawasaki Disease-like features in the setting of recent SARS-CoV-2 infection or exposure [24]. Cases were subsequently reported in New York City and globally, leading the Centers for Disease Control and Prevention (CDC) to issue a national health advisory in May 2020 to alert healthcare providers of multisystem inflammatory syndrome in children (MIS-C) [25,26,27]. The initial case definition for MIS-C included fever, laboratory evidence of inflammation, and evidence of clinically severe illness requiring hospitalization with multisystem organ involvement (e.g., cardiac, renal, respiratory, hematologic, gastrointestinal, dermatologic, or neurologic) without an alternative plausible diagnosis in the setting of recent SARS-CoV-2 infection or exposure in the previous 4 weeks [28]. Accurate identification of cases proved challenging given the broad, evolving case definition of this novel syndrome and the absence of a confirmatory diagnostic test or biomarker. An abundance of studies regarding MIS-C have been published in the past few years, which have greatly broadened our understanding of the epidemiology and management of MIS-C. 

The majority of MIS-C patients are previously healthy, with a median age between 7.3 and 10 years of age [25,29,30,31]. Higher rates of MIS-C are reported in males and in Black and Hispanic children, although this likely reflects the disproportionate impact the COVID-19 pandemic has had on minority communities [2,30]. The most common organ systems involved are gastrointestinal (87%), dermatologic/mucocutaneous (73%), cardiovascular (71%), respiratory (47%), and neurologic (22%) [25,30,32]. Cardiac involvement typically manifests as elevated troponins, brain natriuretic peptides, reduced ejection fraction, pericardial effusion, coronary artery dilation or aneurysm, and arrhythmias [31,33,34]. Laboratory abnormalities include elevated C-reactive protein (CRP), liver enzymes, D-dimer, lymphopenia, neutropenia, thrombocytopenia, and coagulopathy [25,31]. Up to 80% of MIS-C patients require intensive care, approximately half require vasoactive support, 15% require mechanical ventilation, and 2% require extracorporeal membrane oxygenation [25,30]. Risk factors for severe outcomes of MIS-C include older children compared to children 0–5 years of age, non-Hispanic Black patients compared to non-Hispanic White, and patients with increased laboratory abnormalities [35].

Most children with MIS-C recover without long-term sequelae, and the overall mortality rate is low at 1–2% [2,36,37]. In one retrospective cohort study, 57.2% of children with MIS-C had echocardiogram abnormalities at admission, but only 4.7% had persistent abnormalities at a 6-week follow-up [38]. Children with myocarditis due to MIS-C or with myocarditis related to COVID-19 vaccination had faster resolution of cardiac dysfunction compared to a historical cohort of children with viral myocarditis diagnosed prior to the COVID-19 pandemic, with 93% and 100% having normal left ventricular ejection fraction at the time of discharge respectively, compared to 70% in the viral myocarditis cohort [39]. Children with chronic medical conditions prior to hospitalization for MIS-C and greater organ involvement were more likely to report persistent symptoms 2 to 4 months after initial hospitalization and may require more frequent follow-ups [40]. 

Given the broad case definition of MIS-C and overlapping features, distinguishing MIS-C from other hyperinflammatory syndromes, such as Kawasaki Disease and Toxic-Shock syndrome, can be challenging. One multicenter cohort study compared clinical and laboratory characteristics of children admitted with MIS-C, COVID-19, Kawasaki Disease (KD), or Toxic Shock Syndrome (TSS) in order to develop a diagnostic scoring tool to assist in diagnosis [41]. Compared to children with acute COVID-19, KD, or TSS, children with MIS-C had higher rates of cardiac dysfunction, myocarditis, pericardial effusion, and elevated markers of inflammation, cardiac damage, thrombocytopenia, and lymphopenia [41]. In addition, KD tends to impact younger children compared to MIS-C, and gastrointestinal and pulmonary symptoms are more prominent in MIS-C [42,43]. With additional data generated over time, the CDC issued a new MIS-C case definition in December 2022 to more reliably distinguish among MIS-C cases. The updated case definition includes adjustments to the criteria of organ involvement and removes neurologic, renal, and respiratory categories; includes shock as a separate category of organ involvement; utilizes CRP alone as laboratory evidence of inflammation; and classifies cases based on if they are proven, probable, or suspected [44]. 

The optimal treatment of MIS-C remains an area of debate. Given the rarity of MIS-C and the broad case definition, there are no randomized controlled clinical trials comparing treatment regimens for MIS-C. Early in the pandemic, intravenous immunoglobulin (IVIG) was commonly used given the similarity in presentation to Kawasaki Disease, with or without corticosteroids. Based on studies showing the benefit of combination therapy, current NIH COVID-19 treatment recommendations are for IVIG to be used in combination with glucocorticoids for most children hospitalized with MIS-C [45]. A study from the Overcoming COVID-19 surveillance registry comparing IVIG alone to IVIG with glucocorticoids found that combination therapy resulted in a decreased risk for cardiovascular dysfunction and the need for adjunctive immunomodulatory therapy [36]. A few studies have also examined the benefit of monotherapy with IVIG or corticosteroids for the treatment of MIS-C. The Best Available Treatment Study (BATS) was an international observational cohort study of children with MIS-C that utilized propensity-weighted analysis to compare outcomes in children who received combination therapy to either IVIG or glucocorticoid alone [46]. Treatment escalation was less common in children who received combination therapy compared to monotherapy and in children who received glucocorticoids alone compared to children who received IVIG alone. Another retrospective cohort study from four US children’s hospitals also found IVIG and low-dose steroids in combination or low-steroids alone administered within 1 day of hospitalization reduced the risk for prolonged hospitalization [47]. 

MIS-C is, overall, a rare, post-infectious complication of SARS-CoV-2 infection in children [48]. Interestingly, the incidence and severity of MIS-C seemed to decrease with subsequent COVID-19 pandemic waves, potentially due to increased population immunity or viral mutations involved with causing MIS-C [49,50]. In addition, data to date have not shown a significant relationship between COVID-19 vaccination and the development of MIS-C [51]. In fact, vaccination is likely protective against MIS-C, with results from a recent study comparing MIS-C in COVID-19 vaccinated to unvaccinated children demonstrating decreased risk for intensive-care unit admission and mortality in vaccinated children [51]. Although our understanding of MIS-C has greatly improved, much regarding the optimal treatment and exact pathophysiology remains unknown, and continued vigilance is needed given the continued circulation of new SARS-CoV-2 variants. 

## 4. Long COVID-19 

While the vast majority of children and adults with COVID-19 fully recover, a small subset develop persistent, lingering symptoms termed long-COVID-19 or post-COVID-19. Long COVID is defined by the CDC as a broad constellation of symptoms, signs, and conditions that continue or develop after acute COVID-19 infection and are present four weeks or more after the initial phase of infection [52]. Symptoms can include fatigue, post-exertional malaise, fever, respiratory symptoms such as cough or difficulty breathing, neurologic symptoms such as headaches, difficulty concentrating, and gastrointestinal symptoms. Because children and adolescents experience distinct symptoms related to COVID-19 compared to adults, a pediatric-specific clinical case definition of the post-COVID-19 condition was developed by the World Health Organization (WHO) based on expert opinion [53]. The WHO defined long COVID in children as symptoms lasting at least 2 months after confirmed or probable SARS-CoV-2 infection, which generally impact everyday functioning and may be new or persistent, fluctuate, or relapse over time. Similar to adults, children can also present with a wide range of non-specific symptoms, with the most common being altered smell/anosmia, anxiety, fatigue, headache, and loss of appetite [53,54,55]. 

Estimates of the prevalence of long COVID vary widely, likely due to the broad case definition, although it is estimated that over 65 million individuals are affected globally [56]. Data from the National Health Interview Survey in 2022 estimated that 6.9% of US adults reported symptoms consistent with long COVID [57]. Older adults, female sex, severity of acute COVID-19 illness, socioeconomic factors, and comorbidities have been associated with an increased risk for long COVID in adults [57,58]. A retrospective case-control study utilizing data from the NIH Researching COVID to Enhance Recovery (RECOVER) initiative found that hospitalization for COVID-19, the longer duration of hospitalization, and the need for mechanical ventilation were associated with an increased risk for developing long COVID [58]. There is a paucity of pediatric data on long COVID, although similar risk factors have been identified in children. One study of SARS-CoV-2-infected individuals and their household members found that adolescent girls were at higher risk for at least one moderate to severe persistent symptom 11 to 12 months after the initial infection compared to their exposed but uninfected counterparts [59]. A prospective cohort study of children with microbiologically confirmed COVID-19 followed for up to 18 months after infection at a pediatric post-COVID clinic found that pre-existing medical conditions, older age, severity of initial COVID-19 infection, and infection with pre-Omicron variants were risk factors for developing post-COVID symptoms [60]. 

Much remains to be understood about what causes long COVID, who is at risk, and how to identify better, treat, and prevent long COVID. One multi-site cohort study of children who underwent weekly SARS-CoV-2 screening and surveys regarding post-COVID symptoms found that COVID-19 mRNA vaccination was significantly associated with a decreased likelihood of having at least one long-COVID symptom and persistent respiratory symptoms, suggesting vaccination is protective against long-COVID in children [61]. The pathophysiology of long COVID has been postulated to be multifactorial and potentially related to viral persistence, autoimmunity, the reactivation of latent viruses, and chronic inflammatory changes, although further understanding is needed to inform the development of novel diagnostic testing and inform therapeutic development [62]. Results from ongoing studies, including the NIH-sponsored RECOVER initiative, will add to our knowledge of long COVID. 

## 5. Treatment of COVID-19 in Children

As the majority of children remain asymptomatic or minimally symptomatic, most children with COVID-19 infection will only require supportive care. Treatment guidelines have been developed by the National Institutes of Health and the Infectious Diseases Society of America, although the majority of clinical trials on COVID-19 therapeutics have been conducted in adult patients. Thus, guidance for children is generally extrapolated from adult studies [12,45]. The most updated guidance for treatment can be found online from the COVID-19 Treatment Guidelines Panel and is summarized below in Table 1. Children with mild to moderate COVID-19 infection at high risk for progression (e.g., immunosuppressive disease, obesity, medical complexity, neurodevelopmental disorders, severe asthma or chronic lung disease, underlying cardiac disease) to severe disease may benefit from early antiviral therapy with ritonavir-boosted nirmatrelvir (Paxlovid) or remdesivir within 5 or 7 days of symptom onset. A 3-day course of remdesivir decreased the risk of hospitalization by 87% in a randomized, double-blind, placebo-controlled trial of non-hospitalized adult patients at high risk for COVID-19 disease progression [63]. Remdesivir is approved for use by the US Food and Drug Administration (FDA) in children 28 days of age and older weighing at least 3 kg and is currently only administered intravenously, which may limit its use. Ritonavir-boosted nirmatrelvir was demonstrated to decrease the risk of progression to severe COVID-19 by 89% in a phase 2/3 double-blind, randomized trial of non-hospitalized, high-risk adults with symptomatic COVID-19. The emergency use authorization (EUA) for ritonavir-boosted nirmatrelvir is currently approved in the US for children older than 12 years of age and weighing 40 kg or more. In addition, ritonavir is a strong cytochrome P450 inhibitor, and drug interactions should be checked prior to use [64]. 

For children hospitalized with COVID-19 requiring supplemental oxygen, treatment with remdesivir and dexamethasone, alone or in combination, is recommended by expert guidelines, depending on the severity of the illness [45]. For critically ill children, dexamethasone with or without remdesivir is recommended to be started as early as possible. Of note, the safety and efficacy of corticosteroids for the treatment of COVID-19 have not been directly evaluated in children. If children do not have an improvement in oxygenation after dexamethasone, baricitinib or tocilizumab can be considered. Baricitinib is a janus kinase inhibitor approved for emergency use by an FDA EUA for hospitalized children 2 to 17 years of age who require supplemental oxygen, mechanical ventilation, or ECMO. Tocilizumab is an interleukin 6 (IL-6) inhibitor with an FDA EUA for the treatment of hospitalized children older than 2 years of age receiving systemic corticosteroids that require supplemental oxygen and mechanical ventilation of ECMO. 

## 6. Prevention of COVID-19

COVID-19 vaccination is the most important method for preventing COVID-19 infection and its resulting complications. It is estimated that COVID-19 vaccines have prevented 18.5 million hospitalizations and 3.2 million deaths in the US by the end of 2022 [65]. COVID-19 vaccination is recommended for all children six months of age and older without a contraindication. Current FDA-approved vaccines for children include the Pfizer-BioNTech and Moderna mRNA COVID-19 vaccines for children 6 months and older and the Novavax protein subunit vaccine for children 12 years of age and older [65,66]. With the continued circulation of SARS-CoV-2 variants with increased transmissibility and greater antibody escape, there was concern for waning vaccine effectiveness, particularly with the B.1.1.529 Omicron variant [67]. 

The Omicron variants are antigenically distinct from pre-Omicron variants and contain at least 30 mutations in the spike protein [68]. Omicron-specific neutralization titers after vaccination were lower than ancestral strains; however, boosting resulted in improved titers [69,70]. In a prospective cohort study of COVID-19-infected children enrolled when Delta or Omicron BA.1/BA.2 was the predominant circulating strains, post-infection neutralization titers against BA.5 were significantly higher after Omicron infection in children who received two or more vaccine doses compared to unvaccinated children [71]. In addition, updated vaccine effectiveness (VE) data from the CDC showed waning VE against the Omicron XBB variant against hospitalization in adults ≥ 65 years of age between September 2022 and August 2023 [65]. Utilizing a test-negative, case-control design, the vaccine effectiveness of BNT162b2 against COVID-19 hospitalization from laboratory-confirmed infection in children less than 18 years of age was lower during Omicron-predominant circulation (19 December 2021 to 17 February 2022) compared to when the delta variant was predominant (1 July 2021 to 18 December 2021) [72]. But despite waning titers, COVID-19 vaccines remain highly protective against severe outcomes such as hospitalization, need for mechanical ventilation, emergency room visits, and death in children and adults [73,74,75,76]. In a multicenter cohort study of children less than 5 years of age who were admitted to a pediatric hospital due to acute COVID-19, 88.4% of hospitalized children were not vaccinated against COVID-19 despite being eligible, and 7.0% had initiated but did not complete the primary series [77]. Receipt of two doses of BNT162b2 vaccination was still 79% protective against critical COVID-19 illness among adolescents 12 to 18 years of age during omicron [72]. While breakthrough infection can occur, there is strong data to support continued protection against severe outcomes of COVID-19 infection.

Given the data, current CDC recommendations are for all individuals 5 years of age and older to receive one dose of a 2023–2024 updated COVID-19 vaccine at least 8 weeks after their last dose [78]. The updated COVID-19 vaccine more closely targets the XBB.1.5 lineage of the Omicron variant, which accounted for over 99% of circulating strains in the fall of 2023, and has replaced the 2022–2023 Bivalent vaccines that targeted the BA.4 and BA.5 Omicron variants. Children 5 years of age and older who are not vaccinated or have not received an updated COVID-19 vaccine should receive one updated Pfizer-BioNTech or Moderna COVID-19 vaccine. Children 12 years of age and older who are incompletely vaccinated also have the option of receiving the updated Novavax COVID-19 vaccine. Children 6 months to 4 years of age who are not vaccinated should receive three doses of the updated Pfizer-BioNTech vaccine or two doses of the Moderna COVID-19 vaccine. The Johnson & Johnson (New Brunswick, NJ, USA viral vector COVID-19 vaccine is no longer available in the United States as of May 2023 [65,66]. If you have had a COVID-19 infection recently, it is reasonable to delay vaccination by 3 months, given the low risk for re-infection in this time interval. The seasonal influenza vaccine and COVID-19 vaccine are both essential to prevent respiratory viral infections and can be safely administered concomitantly [79]. Given the evolving nature of the pandemic, the most up-to-date COVID-19 vaccine recommendations for the US can be found on the Centers for Disease Control and Prevention website and are summarized in Table 2. 

As of 11 May 2023, over 270 million individuals in the US have received at least one dose of a COVID-19 vaccine [1]. There is an abundance of data to demonstrate the safety of COVID-19 vaccines from both passive and active surveillance systems in the US and globally, such as the US Vaccine Adverse Events Reporting System (VAERS), the Vaccine Safety Datalink (VSD), and the Global Advisory Committee on Vaccine Safety. Safety surveillance data from the VSD of over 247,00 doses of Pfizer-BioNTech or Moderna COVID-19 vaccines administered to children 6 months to less than 5 years of age showed no increased risk for any safety signal during the 21 days after vaccination, and importantly, no cases of myocarditis or pericarditis [80]. In a meta-analysis of studies including children 5 to 11 years of age who received any COVID-19 vaccine, safety data suggested no increased risk of serious adverse events and mainly minor local or systemic reactions that resolved within a few days [81]. Side effects are more common after the second dose of the vaccine. Rare cases of myocarditis and pericarditis after mRNA COVID-19 vaccination have been reported, particularly in male adolescents after receipt of a second mRNA COVID-19 vaccine dose [82]. However, subsequent studies have shown the risk of cardiac complications is significantly higher after COVID-19 infection in both males and females, with a 2–6 times increased relative risk after infection compared to after vaccination in males 12 to 17 years of age [83]. Severe cases of thrombosis with thrombocytopenia syndrome (TTS) were reported after receipt of the J&J/Janssen COVID-19 vaccine, particularly in females less than 50 years of age [84]. The overall rate was low at four cases per one million doses of vaccine administered, and the J&J vaccine is no longer available in the US. Overall, evidence to date strongly supports the safety and benefits of COVID-19 vaccination. 

## 7. Conclusions and Future Directions 

Since the initial descriptions of COVID-19, a substantial number of studies have enhanced our understanding of the epidemiology, clinical presentation, and treatment of COVID-19 infections in children. Children have milder clinical courses with COVID-19 compared to adults; however, a subset can develop severe disease, particularly in children with underlying medical conditions or young infants. Importantly, pediatric providers should remain alert for symptoms and signs consistent with post-infectious complications such as MIS-C and long COVID, which can occur even after mild or asymptomatic infection. The majority of children require only supportive care; however, early antiviral therapy can be considered in high-risk individuals to prevent progression to severe disease. Treatment options for hospitalized children are provided by consensus guidelines, although limited pediatric COVID-19 treatment studies exist. Improved methods of diagnosis and treatment regimens for MIS-C and long COVID are still needed, and ongoing studies will likely yield additional findings in the near future. Finally, with the ongoing emergence of SARS-CoV-2 variants, the optimization of COVID-19 vaccination rates in children should remain a public health priority in this vulnerable population. 

## Figures and Tables

**Table 1:** Guidance for Management of Acute COVID-19 in Children.

Disease Severity	Respiratory Support	Management
Non-hospitalized, regardless of risk	None	Supportive care
Non-hospitalized, high-risk *	None	Age ≥ 12 years Ritonavir-boosted nirmatrelvir (Paxlovid) within 5 days of symptom onset Age ≥ 28 days Remdesivir for 3 days within 7 days of symptom onset
Non-hospitalized, intermediate risk **	None	Insufficient evidence to recommend routine use of antiviral therapy
Hospitalized, high-risk	None	Remdesivir for 5 days or until hospital discharge
Hospitalized, regardless of risk	Conventional oxygen	Use 1 of the following: Remdesivir for 5 days or until hospital discharge Dexamethasone plus remdesivir for children with increasing oxygen needs
	High-flow or Non-invasive ventilation oxygen	Use 1 of the following: Dexamethasone Dexamethasone plus remdesivir
	Mechanical ventilation or ECMO	Dexamethasone Consider remdesivir, benefit is unclear Consider baricitinib or tocilizumab for children who do not have rapid improvement in oxygenation

Adapted from the NIH COVID-19 Treatment Guideline. * High-risk conditions include individuals moderately or severely immunocompromised, children with any of the following conditions: obesity, medically complex with dependence on respiratory technology; severe neurologic, genetic, metabolic, or other factors that impair airway clearance; severe asthma or chronic lung disease; severe congenital or acquired cardiac disease; and multiple moderate to severe chronic diseases, particularly if unvaccinated. ** Intermediate-risk conditions: children less than 1 year of age; prematurity in children less than 2 years of age; sickle cell disease; poorly controlled diabetes mellitus; and non-severe cardiac, neurologic, or metabolic disease.

**Table 2:** Current CDC Recommendations for Updated 2023–2024 COVID-19 Vaccines in Healthy Children.

Age	Updated Vaccine Product 2023–2024	Primary Series	Booster
6 months to 4 years	Moderna	Two doses, 4–8 weeks apart	One dose of updated vaccine if received 1+ dose of any Moderna vaccine, >8 weeks after last dose
	Pfizer-BioNTech	Three doses, 3–8 weeks after first dose, >8 weeks after second dose	Two doses of updated vaccine if received one dose of any Pfizer vaccine, 3–8 weeks after first dose, >8 weeks after second dose One dose of updated vaccine if received two or more doses of any Pfizer vaccine, >8 weeks after last dose
5 years to 11 years	Moderna	One dose	One dose of any updated mRNA vaccine
	Pfizer-BioNTech	One dose	One dose of any updated mRNA vaccine
12 years and older	Moderna	One dose	One dose of updated vaccine if received one or more doses of any mRNA, Novavax, or Janssen vaccine doses
	Pfizer-BioNTech	One dose	One dose of updated vaccine if received one or more doses of any mRNA, Novavax, or Janssen vaccine doses
	Novavax	Two doses, >8 weeks after first dose	One dose of updated vaccine if received one or more doses of any mRNA, Novavax, or Janssen vaccine doses

Adapted from the CDC Website Stay Up to Date with COVID-19 Vaccines.

## References

[1] CDC (2023). COVID Data Tracker. https://covid.cdc.gov/covid-data-tracker.

[2] Blatz A.M., Randolph A.G. (2022). Severe COVID-19 and Multisystem Inflammatory Syndrome in Children in Children and Adolescents. Crit. Care Clin..

[3] Bi Q., Wu Y., Mei S., Ye C., Zou X., Zhang Z., Liu X., Wei L., Truelove S.A., Zhang T. (2020). Epidemiology and transmission of COVID-19 in 391 cases and 1286 of their close contacts in Shenzhen, China: A retrospective cohort study. Lancet Infect. Dis..

[4] Dawood F.S., Porucznik C.A., Veguilla V., Stanford J.B., Duque J., Rolfes M.A., Dixon A., Thind P., Hacker E., Castro M.J.E. (2022). Incidence Rates, Household Infection Risk, and Clinical Characteristics of SARS-CoV-2 Infection among Children and Adults in Utah and New York City, New York. JAMA Pediatr..

[5] Chu V.T., Yousaf A.R., Chang K., Schwartz N.G., McDaniel C.J., Lee S.H., Szablewski C.M., Brown M., Drenzek C.L., Dirlikov E. (2021). Household Transmission of SARS-CoV-2 from Children and Adolescents. N. Engl. J. Med..

[6] Paul L.A., Daneman N., Schwartz K.L., Science M., Brown K.A., Whelan M., Chan E., Buchan S.A. (2021). Association of Age and Pediatric Household Transmission of SARS-CoV-2 Infection. JAMA Pediatr..

[7] Boutzoukas A.E., Zimmerman K.O., Inkelas M., Brookhart M.A., Benjamin D.K., Butteris S., Koval S., DeMuri G.P., Manuel V.G., Smith M.J. (2022). School Masking Policies and Secondary SARS-CoV-2 Transmission. Pediatrics.

[8] Dawson P., Worrell M.C., Malone S., Fritz S.A., McLaughlin H.P., Montgomery B.K., Boyle M., Gomel A., Hayes S., Maricque B. (2022). Modifications to student quarantine policies in K-12 schools implementing multiple COVID-19 prevention strategies restores in-person education without increasing SARS-CoV-2 transmission risk, January–March 2021. PLoS ONE.

[9] Thakkar P.V., Zimmerman K.O., Brookhart M.A., Erickson T.R., Benjamin D.K., Kalu I.C., ABC Science Collaborative (2022). COVID-19 Incidence Among Sixth through Twelfth Grade Students by Vaccination Status. Pediatrics.

[10] Campbell M.M., Benjamin D.K., Mann T.K., Fist A., Blakemore A., Diaz K.S., Kim H., Edwards L.J., Rak Z., Brookhart M.A. (2022). Test-to-Stay After SARS-CoV-2 Exposure: A Mitigation Strategy for Optionally Masked K-12 Schools. Pediatrics.

[11] Karron R.A., Hetrich M.K., Na Y.B., Knoll M.D., Schappell E., Meece J., Hanson E., Tong S., Lee J.S., Veguilla V. (2022). Assessment of Clinical and Virological Characteristics of SARS-CoV-2 Infection among Children Aged 0 to 4 Years and Their Household Members. JAMA Netw. Open.

[12] Hayden M.K., Hanson K.E., Englund J.A., Lee F., Lee M.J., Loeb M., Morgan D.J., Patel R., El Alayli A., El Mikati I.K. (2023). The Infectious Diseases Society of America Guidelines on the Diagnosis of COVID-19: Antigen Testing. Clin. Infect. Dis..

[13] Inagaki K., Hobbs C.V. (2023). COVID-19: A Pediatric Update in Epidemiology, Management, Prevention, and Long-term Effects. Pediatr Rev.

[14] Christophers B., Gallo Marin B., Oliva R., Powell W.T., Savage T.J., Michelow I.C. (2022). Trends in clinical presentation of children with COVID-19: A systematic review of individual participant data. Pediatr. Res..

[15] Hoang A., Chorath K., Moreira A., Evans M., Burmeister-Morton F., Burmeister F., Naqvi R., Petershack M., Moreira A. (2020). COVID-19 in 7780 pediatric patients: A systematic review. EClinicalMedicine.

[16] Woodruff R.C., Campbell A.P., Taylor C.A., Chai S.J., Kawasaki B., Meek J., Anderson E.J., Weigel A., Monroe M.L., Reeg L. (2022). Risk Factors for Severe COVID-19 in Children. Pediatrics.

[17] Flaxman S., Whittaker C., Semenova E., Rashid T., Parks R.M., Blenkinsop A., Unwin H.J.T., Mishra S., Bhatt S., Gurdasani D. (2023). Assessment of COVID-19 as the Underlying Cause of Death Among Children and Young People Aged 0 to 19 Years in the US. JAMA Netw. Open.

[18] Hobbs C.V., Woodworth K., Young C.C., Jackson A.M., Newhams M.M., Dapul H., Maamari M., Hall M.W., Maddux A.B., Singh A.R. (2022). Frequency, Characteristics and Complications of COVID-19 in Hospitalized Infants. Pediatr. Infect. Dis. J..

[19] Smith N., Possémé C., Bondet V., Sugrue J., Townsend L., Charbit B., Rouilly V., Saint-André V., Dott T., Pozo A.R. (2022). Defective activation and regulation of type I interferon immunity is associated with increasing COVID-19 severity. Nat. Commun..

[20] Brodin P. (2022). SARS-CoV-2 infections in children: Understanding diverse outcomes. Immunity.

[21] Halasa N.B., Spieker A.J., Young C.C., Olson S.M., Newhams M.M., Amarin J.Z., Moffitt K.L., Nakamura M.M., Levy E.R., Soma V.L. (2023). Life-Threatening Complications of Influenza vs Coronavirus Disease 2019 (COVID-19) in US Children. Clin. Infect. Dis..

[22] Jone P.N., John A., Oster M.E., Allen K., Tremoulet A.H., Saarel E.V., Lambert L.M., Miyamoto S.D., de Ferranti S.D. (2022). SARS-CoV-2 Infection and Associated Cardiovascular Manifestations and Complications in Children and Young Adults: A Scientific Statement from the American Heart Association. Circulation.

[23] LaRovere K.L., Riggs B.J., Poussaint T.Y., Young C.C., Newhams M.M., Maamari M., Walker T.C., Singh A.R., Dapul H., Hobbs C.V. (2021). Neurologic Involvement in Children and Adolescents Hospitalized in the United States for COVID-19 or Multisystem Inflammatory Syndrome. JAMA Neurol..

[24] Riphagen S., Gomez X., Gonzalez-Martinez C., Wilkinson N., Theocharis P. (2020). Hyperinflammatory shock in children during COVID-19 pandemic. Lancet.

[25] Feldstein L.R., Rose E.B., Horwitz S.M., Collins J.P., Newhams M.M., Son M.B.F., Newburger J.W., Kleinman L.C., Heidemann S.M., Martin A.A. (2020). Multisystem Inflammatory Syndrome in U. S. Children and Adolescents. N. Engl. J. Med..

[26] Verdoni L., Mazza A., Gervasoni A., Martelli L., Ruggeri M., Ciuffreda M., Bonanomi E., D’Antiga L. (2020). An outbreak of severe Kawasaki-like disease at the Italian epicentre of the SARS-CoV-2 epidemic: An observational cohort study. Lancet.

[27] Dufort E.M., Koumans E.H., Chow E.J., Rosenthal E.M., Muse A., Rowlands J., Barranco M.A., Maxted A.M., Rosenberg E.S., Easton D. (2020). Multisystem Inflammatory Syndrome in Children in New York State. N. Engl. J. Med..

[28] CDC, Multisystem Inflammatory Syndrome in Children (MIS-C) Associated with Coronavirus Disease 2019 (COVID-19). 2020: CDC Health Alert Network. https://emergency.cdc.gov/han/2020/han00431.asp.

[29] Godfred-Cato S., Bryant B., Leung J., Oster M.E., Conklin L., Abrams J., Roguski K., Wallace B., Prezzato E., Koumans E.H. (2020). COVID-19-Associated Multisystem Inflammatory Syndrome in Children—United States, March–July 2020. MMWR Morb. Mortal Wkly. Rep..

[30] Abrams J.Y., Godfred-Cato S.E., Oster M.E., Chow E.J., Koumans E.H., Bryant B., Leung J.W., Belay E.D. (2020). Multisystem Inflammatory Syndrome in Children Associated with Severe Acute Respiratory Syndrome Coronavirus 2: A Systematic Review. J. Pediatr..

[31] Dionne A., Son M.B.F., Randolph A.G. (2022). An Update on Multisystem Inflammatory Syndrome in Children Related to SARS-CoV-2. Pediatr. Infect. Dis. J..

[32] Belay E.D., Abrams J., Oster M.E., Giovanni J., Pierce T., Meng L., Prezzato E., Balachandran N., Openshaw J.J., Rosen H.E. (2021). Trends in Geographic and Temporal Distribution of US Children with Multisystem Inflammatory Syndrome during the COVID-19 Pandemic. JAMA Pediatr..

[33] Hensley M., Goodman M., Madani R., Jaggi P., Keesari R., Zhang Q., Oster M.E. (2023). Cardiac complications in children with acute COVID-19 vs multisystem inflammatory syndrome in children (MIS-C). Am. Heart J..

[34] Alsaied T., Tremoulet A.H., Burns J.C., Saidi A., Dionne A., Lang S.M., Newburger J.W., de Ferranti S., Friedman K.G. (2021). Review of Cardiac Involvement in Multisystem Inflammatory Syndrome in Children. Circulation.

[35] Abrams J.Y., Oster M.E., Godfred-Cato S.E., Bryant B., Datta S.D., Campbell A.P., Leung J.W., Tsang C.A., Pierce T.J., Kennedy J.L. (2021). Factors linked to severe outcomes in multisystem inflammatory syndrome in children (MIS-C) in the USA: A retrospective surveillance study. Lancet Child Adolesc. Health.

[36] Son M.B.F., Murray N., Friedman K., Young C.C., Newhams M.M., Feldstein L.R., Loftis L.L., Tarquinio K.M., Singh A.R., Heidemann S.M. (2021). Multisystem Inflammatory Syndrome in Children—Initial Therapy and Outcomes. N. Engl. J. Med..

[37] Feldstein L.R., Tenforde M.W., Friedman K.G., Newhams M., Rose E.B., Dapul H., Soma V.L., Maddux A.B., Mourani P.M., Bowens C. (2021). Characteristics and Outcomes of US Children and Adolescents with Multisystem Inflammatory Syndrome in Children (MIS-C) Compared with Severe Acute COVID-19. JAMA.

[38] Kaltman J., Keesari R., Madani R., Jaggi P., Oster M.E. (2023). Six-month cardiac outcomes in children with multisystem inflammatory syndrome in children. Cardiol. Young.

[39] Patel T., Kelleman M., West Z., Peter A., Dove M., Butto A., Oster M.E. (2022). Comparison of Multisystem Inflammatory Syndrome in Children-Related Myocarditis, Classic Viral Myocarditis, and COVID-19 Vaccine-Related Myocarditis in Children. J. Am. Heart. Assoc..

[40] Maddux A.B., Young C.C., Kucukak S., Zambrano L.D., Newhams M.M., Rollins C.K., Halasa N.B., Gertz S.J., Mack E.H., Schwartz S. (2023). Risk factors for health impairments in children after hospitalization for acute COVID-19 or MIS-C. Front. Pediatr..

[41] Godfred-Cato S., Abrams J.Y., Balachandran N., Jaggi P., Jones K., Rostad C.A., Lu A.T., Fan L., Jabbar A., Anderson E.J. (2022). Distinguishing Multisystem Inflammatory Syndrome in Children from COVID-19, Kawasaki Disease and Toxic Shock Syndrome. Pediatr. Infect. Dis. J..

[42] Wessels P.A., Bingler M.A. (2022). A comparison of Kawasaki Disease and multisystem inflammatory syndrome in children. Prog. Pediatr. Cardiol..

[43] Fan L.K., Bai S., Du C., Bass M., Jones K., Sherry W., Morris C.R., Oster M.E., Shane A.L., Jaggi P. (2023). Distinguishing Incomplete Kawasaki and Nonsevere Multisystem Inflammatory Syndrome in Children. Hosp. Pediatr..

[44] Melgar M., Lee E.H., Miller A.D., Lim S., Brown C.M., Yousaf A.R., Zambrano L.D., Belay E.D., Godfred-Cato S., Abrams J.Y. (2022). Council of State and Territorial Epidemiologists/CDC Surveillance Case Definition for Multisystem Inflammatory Syndrome in Children Associated with SARS-CoV-2 Infection—United States. MMWR Recomm. Rep..

[45] NIH (2023). COVID-19 Treatment Guidelines Panel: Coronavirus Disease 2019 (COVID-19) Treatment Guidelines. https://www.covid19treatmentguidelines.nih.gov/.

[46] Channon-Wells S., Vito O., McArdle A.J., Seaby E.G., Patel H., Shah P., Pazukhina E., Wilson C., Broderick C., D’Souza G. (2023). Immunoglobulin, glucocorticoid, or combination therapy for multisystem inflammatory syndrome in children: A propensity-weighted cohort study. Lancet Rheumatol..

[47] Shah A.B., Abrams J.Y., Godfred-Cato S., Kunkel A., Hammett T.A., Perez M.A., Hsiao H.M., Baida N., Rostad C.A., Ballan W. (2023). Treatments and Severe Outcomes for Patients Diagnosed with MIS-C at Four Children’s Hospitals in the United States, March 16, 2020-March 10, 2021. Pediatr. Infect. Dis. J..

[48] Payne A.B., Gilani Z., Godfred-Cato S., Belay E.D., Feldstein L.R., Patel M.M., Randolph A.G., Newhams M., Thomas D., Magleby R. (2021). Incidence of Multisystem Inflammatory Syndrome in Children among US Persons Infected with SARS-CoV-2. JAMA Netw. Open.

[49] Cohen J.M., Carter M.J., Cheung C.R., Ladhani S. (2023). Lower Risk of Multisystem Inflammatory Syndrome in Children with the Delta and Omicron Variants of Severe Acute Respiratory Syndrome Coronavirus 2. Clin. Infect. Dis..

[50] Miller A.D., Yousaf A.R., Bornstein E., Wu M.J., Lindsey K., Melgar M., Oster M.E., Zambrano L.D., Campbell A.P. (2022). Multisystem Inflammatory Syndrome in Children during Severe Acute Respiratory Syndrome Coronavirus 2 (SARS-CoV-2) Delta and Omicron Variant Circulation-United States, July 2021–January 2022. Clin. Infect. Dis..

[51] Yousaf A.R., Miller A.D., Lindsey K., Shah A.B., Wu M.J., Melgar M., Zambrano L.D., Campbell A.P. (2023). Multisystem Inflammatory Syndrome in Children among Persons Who Completed a Two-dose COVID-19 Vaccine Primary Series Compared with Those Reporting No COVID-19 Vaccination, US National MIS-C Surveillance. Pediatr. Infect. Dis. J..

[52] CDC (2023). Long COVID or Post-COVID Conditions. 2023. https://www.cdc.gov/coronavirus/2019-ncov/long-term-effects/index.html.

[53] WHO (2023). A clinical Case Definition for Post COVID-19 Condition in Children and Adolescents by Expert Consensus, 16 February 2023. https://www.who.int/publications/i/item/WHO-2019-nCoV-Post-COVID-19-condition-CA-Clinical-case-definition-2023-1.

[54] Lopez-Leon S., Wegman-Ostrosky T., Ayuzo Del Valle N.C., Perelman C., Sepulveda R., Rebolledo P.A., Cuapio A., Villapol S. (2022). Long-COVID in children and adolescents: A systematic review and meta-analyses. Sci. Rep..

[55] Rao S., Lee G.M., Razzaghi H., Lorman V., Mejias A., Pajor N.M., Thacker D., Webb R., Dickinson K., Bailey L.C. (2022). Clinical Features and Burden of Postacute Sequelae of SARS-CoV-2 Infection in Children and Adolescents. JAMA Pediatr..

[56] Davis H.E., McCorkell L., Vogel J.M., Topol E.J. (2023). Long COVID: Major findings, mechanisms and recommendations. Nat. Rev. Microbiol..

[57] Adjaye-Gbewonyo D., Vahratian A., Perrine C.G., Bertolli J. (2023). Long COVID in Adults: United States, 2022.

[58] Hill E.L., Mehta H.B., Sharma S., Mane K., Singh S.K., Xie C., Cathey E., Loomba J., Russell S., Spratt H. (2023). Risk factors associated with post-acute sequelae of SARS-CoV-2: An N3C and NIH RECOVER study. BMC Public Health.

[59] Haddad A., Janda A., Renk H., Stich M., Frieh P., Kaier K., Lohrmann F., Nieters A., Willems A., Huzly D. (2022). Long COVID symptoms in exposed and infected children, adolescents and their parents one year after SARS-CoV-2 infection: A prospective observational cohort study. EBioMedicine.

[60] Morello R., Mariani F., Mastrantoni L., De Rose C., Zampino G., Munblit D., Sigfrid L., Valentini P., Buonsenso D. (2023). Risk factors for post-COVID-19 condition (Long Covid) in children: A prospective cohort study. EClinicalMedicine.

[61] Yousaf A.R., Mak J., Gwynn L., Bloodworth R., Rai R., Jeddy Z., LeClair L.B., Edwards L., Olsho L.E.W., Newes-Adeyi G. (2023). COVID-19 mRNA Vaccination Reduces the Occurrence of Post-COVID Conditions in U.S. Children Aged 5-17 Years Following Omicron SARS-CoV-2 Infection, July 2021–September 2022. Open Forum Infect. Dis..

[62] Iwasaki A., Putrino D. (2023). Why we need a deeper understanding of the pathophysiology of long COVID. Lancet Infect. Dis..

[63] Gottlieb R.L., Vaca C.E., Paredes R., Mera J., Webb B.J., Perez G., Oguchi G., Ryan P., Nielsen B.U., Brown M. (2022). Early Remdesivir to Prevent Progression to Severe COVID-19 in Outpatients. N. Engl. J. Med..

[64] Hammond J., Leister-Tebbe H., Gardner A., Abreu P., Bao W., Wisemandle W., Baniecki M., Hendrick V.M., Damle B., Simón-Campos A. (2022). Oral Nirmatrelvir for High-Risk, Nonhospitalized Adults with COVID-19. N. Engl. J. Med..

[65] Regan J.J., Moulia D.L., Link-Gelles R., Godfrey M., Mak J., Najdowski M., Rosenblum H.G., Shah M.M., Twentyman E., Meyer S. (2023). Use of Updated COVID-19 Vaccines 2023–2024 Formula for Persons Aged ≥6 Months: Recommendations of the Advisory Committee on Immunization Practices—United States, September 2023. MMWR Morb. Mortal Wkly. Rep..

[66] CDC (2023). Overview of COVID-19 Vaccines. https://www.cdc.gov/coronavirus/2019-ncov/vaccines/different-vaccines/overview-COVID-19-vaccines.html.

[67] Barouch D.H. (2022). COVID-19 Vaccines–Immunity, Variants, Boosters. N. Engl. J. Med..

[68] Dejnirattisai W., Huo J., Zhou D., Zahradník J., Supasa P., Liu C., Duyvesteyn H.M.E., Ginn H.M., Mentzer A.J., Tuekprakhon A. (2022). SARS-CoV-2 Omicron-B.1.1.529 leads to widespread escape from neutralizing antibody responses. Cell.

[69] Sievers B.L., Chakraborty S., Xue Y., Gelbart T., Gonzalez J.C., Cassidy A.G., Golan Y., Prahl M., Gaw S.L., Arunachalam P.S. (2022). Antibodies elicited by SARS-CoV-2 infection or mRNA vaccines have reduced neutralizing activity against Beta and Omicron pseudoviruses. Sci. Transl. Med..

[70] Lassaunière R., Polacek C., Frische A., Boding L., Sækmose S.G., Rasmussen M., Fomsgaard A. (2022). Neutralizing Antibodies Against the SARS-CoV-2 Omicron Variant (BA.1) 1 to 18 Weeks After the Second and Third Doses of the BNT162b2 mRNA Vaccine. JAMA Netw. Open.

[71] Belongia E.A., Petrie J.G., Feldstein L.R., Guan L., Halfmann P.J., King J.P., Neumann G., Pattinson D., Rolfes M.A., McLean H.Q. (2023). Neutralizing Immunity Against Antigenically Advanced Omicron BA.5 in Children After SARS-CoV-2 Infection. J. Pediatr. Infect. Dis. Soc..

[72] Price A.M., Olson S.M., Newhams M.M., Halasa N.B., Boom J.A., Sahni L.C., Pannaraj P.S., Irby K., Bline K.E., Maddux A.B. (2022). Overcoming Covid-19 Investigators. BNT162b2 Protection against the Omicron Variant in Children and Adolescents. N. Engl. J. Med..

[73] Link-Gelles R., Ciesla A.A., Rowley E.A.K., Klein N.P., Naleway A.L., Payne A.B., Kharbanda A., Natarajan K., DeSilva M.B., Dascomb K. (2023). Effectiveness of Monovalent and Bivalent mRNA Vaccines in Preventing COVID-19-Associated Emergency Department and Urgent Care Encounters among Children Aged 6 Months-5 Years—VISION Network, United States, July 2022–June 2023. MMWR Morb. Mortal Wkly. Rep..

[74] Gray G., Collie S., Goga A., Garrett N., Champion J., Seocharan I., Bamford L., Moultrie H., Bekker L.G. (2022). Effectiveness of Ad26.COV2.S and BNT162b2 Vaccines against Omicron Variant in South Africa. N. Engl. J. Med..

[75] Britton A., Embi P.J., Levy M.E., Gaglani M., DeSilva M.B., Dixon B.E., Dascomb K., Patel P., Schrader K.E., Klein N.P. (2022). Effectiveness of COVID-19 mRNA Vaccines Against COVID-19-Associated Hospitalizations among Immunocompromised Adults during SARS-CoV-2 Omicron Predominance—VISION Network, 10 States, December 2021–August 2022. MMWR Morb. Mortal Wkly. Rep..

[76] DeCuir J., Surie D., Zhu Y., Gaglani M., Ginde A.A., Douin D.J., Talbot H.K., Casey J.D., Mohr N.M., McNeal T. (2023). Effectiveness of Monovalent mRNACOVID-19 Vaccination in Preventing COVID-19-Associated Invasive Mechanical Ventilation Death among Immunocompetent Adults during the Omicron Variant Period—IVYNetwork 19, U.S. States, February 1, 2022–January 31, 2023. MMWR Morb. Mortal Wkly. Rep..

[77] Zambrano L.D., Newhams M.M., Simeone R.M., Fleming-Dutra K.E., Halasa N., Wu M., Orzel-Lockwood A.O., Kamidani S., Pannaraj P.S., Chiotos K. (2023). Characteristics and Clinical Outcomes of Vaccine-Eligible US Children Under-5 Years Hospitalized for Acute COVID-19 in a National Network. Pediatr. Infect. Dis. J..

[78] Moulia D.L., Wallace M., Roper L.E., Godfrey M., Rosenblum H.G., Link-Gelles R., Britton A., Daley M.F., Meyer S., Fleming-Dutra K.E. (2023). Interim Recommendations for Use of Bivalent mRNA COVID-19 Vaccines for Persons Aged ≥6 Months—United States, April 2023. MMWR Morb. Mortal Wkly. Rep..

[79] Hause A.M., Zhang B., Yue X., Marquez P., Myers T.R., Parker C., Gee J., Su J., Shimabukuro T.T., Shay D.K. (2022). Reactogenicity of Simultaneous COVID-19 mRNA Booster and Influenza Vaccination in the US. JAMA Netw Open.

[80] Goddard K., Donahue J.G., Lewis N., Hanson K.E., Weintraub E.S., Fireman B., Klein N.P. (2023). Safety of COVID-19 mRNA Vaccination among Young Children in the Vaccine Safety Datalink. Pediatrics.

[81] Piechotta V., Siemens W., Thielemann I., Toews M., Koch J., Vygen-Bonnet S., Kothari K., Grummich K., Braun C., Kapp P. (2023). Safety and effectiveness of vaccines against COVID-19 in children aged 5–11 years: A systematic review and meta-analysis. Lancet Child Adolesc. Health.

[82] Oster M.E., Shay D.K., Su J.R., Gee J., Creech C.B., Broder K.R., Edwards K., Soslow J.H., Dendy J.M., Schlaudecker E. (2022). Myocarditis Cases Reported After mRNA-Based COVID-19 Vaccination in the US from December 2020 to August 2021. JAMA.

[83] Block J.P., Boehmer T.K., Forrest C.B., Carton T.W., Lee G.M., Ajani U.A., Christakis D.A., Cowell L.G., Draper C., Ghildayal N. (2022). Cardiac Complications After SARS-CoV-2 Infection and mRNA COVID-19 Vaccination—PCORnet, United States, January 2021–January 2022. MMWR Morb. Mortal Wkly. Rep..

[84] See I., Lale A., Marquez P., Streiff M.B., Wheeler A.P., Tepper N.K., Woo E.J., Broder K.R., Edwards K.M., Gallego R. (2022). Case Series of Thrombosis with Thrombocytopenia Syndrome After COVID-19 Vaccination-United States, December 2020 to August 2021. Ann. Intern. Med..

